# A Comparison Study of Single-Echo Susceptibility Weighted Imaging and Combined Multi-Echo Susceptibility Weighted Imaging in Visualizing Asymmetric Medullary Veins in Stroke Patients

**DOI:** 10.1371/journal.pone.0159251

**Published:** 2016-08-05

**Authors:** Chao Wang, Tiantian Qiu, Ruirui Song, Yerfan Jiaerken, Linglin Yang, Shaoze Wang, Minming Zhang, Xinfeng Yu

**Affiliations:** 1 Department of Radiology, the Second Affiliated Hospital, Zhejiang University School of Medicine, Hangzhou, China; 2 Department of Neurology, the Second Affiliated Hospital, Zhejiang University School of Medicine, Hangzhou, China; 3 Department of Electrical Engineering, Zhejiang University, Hangzhou, China; Fraunhofer Research Institution of Marine Biotechnology, GERMANY

## Abstract

**Background:**

Asymmetric medullary veins (AMV) are frequently observed in stroke patients and single-echo susceptibility weighted imaging (SWI_s_) is the main technique in detecting AMV. Our study aimed to investigate which echo time (TE) on single-echo susceptibility is the optimal echo for visualizing AMV and to compare the ability in detecting AMV in stroke patients between SWI_s_ and multi-echo susceptibility weighted imaging (SWI_c_).

**Materials and Methods:**

Twenty patients with middle cerebral artery stroke were included. SWI was acquired by using a multi-echo gradient-echo sequence with six echoes ranging from 5 ms to 35.240 ms. Three different echoes of SWI_s_ including SWI_s1_ (TE = 23.144 ms), SWI_s2_ (TE = 29.192 ms) and SWI_s3_ (TE = 35.240 ms) were reconstructed. SWI_c_ was averaged using the three echoes of SWI_s_. Image quality and venous contrast of medullary veins were compared between SWI_s_ and SWI_c_ using peak signal-to-noise ratio (PSNR), mean opinion score (MOS), contrast-to-noise ratio (CNR) and signal-to-noise ratio (SNR). The presence of AMV was evaluated in each SWI_s (1–3)_ and SWI_c_.

**Results:**

SWI_s2_ had the highest PSNR, MOS and CNR and SWI_s1_ had the highest SNR among three different echoes of SWI_s_. No significant difference was found in SNR between SWI_s1_ and SWI_s2_. PSNR, MOS and CNR in SWI_c_ were significantly increased by 27.9%, 28.2% and 17.2% compared with SWI_s2_ and SNR in SWI_c_ was significantly increased by 32.4% compared with SWI_s1_. 55% of patients with AMV were detected in SWI_s2_, SWI_s3_ and SWI_c_, while 50% AMV were found in SWI_s1_.

**Conclusions:**

SWI_s_ using TE around 29ms was optimal in visualizing AMV. SWI_c_ could improve image quality and venous contrast, but was equal to SWI_s_ using a relative long TE in evaluating AMV. These results provide the technique basis for further research of AMV in stroke.

## Introduction

Susceptibility weighted imaging (SWI) is a high spatial resolution three dimensional (3D) gradient echo magnetic resonance imaging (MRI) technique that exploits the differences in magnetic susceptibility of various tissues, such as blood products, iron, and calcification [1–3]. In previous studies, we have reported that phase values from single-echo SWI (SWI_s_) and R2* values derived from combined multi-echo SWI (SWI_c_) are useful imaging biomarkers of brain iron deposition in vivo [4, 5]. In addition to quantitative measurement of brain iron, SWI is also a noninvasive means which can visualize artery thrombi and venous system in the brain without contrast agent injection [6, 7].

The cerebral venous architecture, especially deep medullary veins (DMV) at the periventricular white matter areas of the cerebral hemispheres, has recently been paid increased attention because these small veins have important clinical and pathophysiological significance in cerebrovascular diseases [8–11]. Asymmetric medullary veins (AMV) are frequently detected in stroke patients [12, 13], and has been reported to be associated with development of hemorrhagic transformation in patients with acute stroke treated with intravenous tissue plasminogen activator [9]. Because of the small diameter of DMV, it is essential to choose appropriate technique to depict DMV clearly. If there were vague DMV in MR images, it would be difficult to determine the presence of AMV.

SWI_s_ using a specific echo time (TE) remains the main SWI technique in clinical stroke research [14]. It has been reported that optimal venous contrast of a vein parallel to the main field is achieved at TE = 28 ms [15, 16]. However, which TE would produce the best image contrast of DMV which are perpendicular to the main field is still unknown. On the other hand, SWI_c_ images are proposed to have better contrast-to-noise ratio (CNR) and signal-to-noise ratio (SNR) than SWI_s_ images [17]. Furthermore, different echoes can be acquired simultaneously within one repetition time (TR) period without increasing scan time [18]. Recently, several papers have focused on the value of SWI in detecting AMV [8, 19], but few studies have been previously published about the comparison of SWI_s_ and SWI_c_ in visualizing AMV. In our study, we used a high resolution multi-echo SWI sequence to analyze which TE yields the optimum image quality and venous contrast of DMV for identifying AMV in a subset of stroke patients. At the meantime, we compared the image quality and venous contrast of DMV in SWI_c_ with those in SWI_s_ and compared the ability of SWI_c_ and SWI_s_ in detecting AMV.

## Materials and Methods

### Patients

The study was approved by the human ethics committee of the second affiliated hospital, Zhejiang University School of Medicine (No.2013LSY-006) and written informed consent was obtained from all subjects. Every participant was informed of the purpose and procedure of this study.

Twenty patients with middle cerebral artery (MCA) territory infarction within 3 to 7 days after symptom onset between April 2013 and January 2014 were recruited. All patients did not receive any thrombolytic or recanalization therapies. The inclusion criteria were as follows: 1) first-ever ischemic stroke; 2) involving the vascular territory of unilateral MCA; 3) admission between 3 and 7 days after stroke onset; 4) age older than 18 years; 5) without hemorrhagic infarction; 6) without history of neurological or psychiatric disorders.

### Data acquisition

Each patient was scanned using a 3D high resolution flow-compensated multi-echo SWI sequence on a 3.0T MR scanner (Signa Excite, GE Healthcare, USA) by using an 8-channel brain phased array coil. The detail parameters of SWI sequence were as follows: TR = 45 ms, TE = 5 ms to 35.240 ms with an echo spacing of 6.048ms, echo number = 6, flip angle = 25°, bandwidth (BW) = 41.67 kHz, slice thickness = 2 mm, matrix = 384 × 320, field of view (FOV) = 24 cm. To further improve in-plane resolution, 384 × 320 images were interpolated into 512 × 512 images, which yielded an in-plane resolution of 0.47 × 0.47 mm^2^. In order to save scan time, scan covering supratentorial regions was performed. Brain images were collected parallel to anterior commissure–posterior commissure line. The total acquisition time was 4 min 25 sec.

### SWI process

We used the latter three echoes (including TE of 23.144 ms, 29.192 ms, and 35.240 ms) from multi-echo SWI sequence to reconstruct three different echoes of SWI_s_. Each echo of SWI_s_ was processed based on magnitude and phase images using the SPIN software (Signal Processing in NMR, Detroit, Michigan, USA). The process involves high pass filter with 64 × 64, phase multiplication with factor of 4 and minimum intensity projection (mIP) over 4 slices. Lastly, three different echoes of SWI_s_ including SWI_s1_ (TE = 23.144 ms), SWI_s2_ (TE = 29.192 ms), SWI_s3_ (TE = 35.240 ms) were obtained. Then, SWI_c_ was processed by averaging the three different echoes of SWI_s_ [17].

### SWI image analysis

#### 1. Image quality comparison

**a. Objective comparison:** Peak signal-to-noise ratio (PSNR) of SWI_s1_, SWI_s2_, SWI_s3_ and SWI_c_ images were calculated to analytically compare image quality using MATLAB software.

The PSNR was defined as follows [20]:

$$Mean\;squared\;error\;\left(MSE\right)=\overset{}{\underset{}{\sum_{i=1}^{x}\sum_{j=1}^{y}\frac{{\left(\left|A_{ij}-B_{ij}\right|\right)}^{2}}{x\cdot y}}}$$ (1)

$$PSNR=10\cdot \text{log}\left(\frac{255^{2}}{MSE}\right)$$ (2)

where A_ij_ is the original image data and B_ij_ is the compressed image value and x × y represents image size. The units of PSNR are in decibels (dB) and higher PSNR indicates better image quality.

**b. Subjective comparison:** Image quality was also analyzed by subjective method of mean opinion score (MOS) [21] with five-point scale by two radiologists who were blinded to the study goal and gave a consensus result. Each type of SWI images were evaluated at an interval of one week using the same window level and window width by SPIN software. The five-point scale is as follows: a score of 5 indicates excellent; a score of 4, good; a score of 3, moderate; a score of 2, poor; and a score of 1, nondiagnostic [22]. Inter-rater reliability was analyzed using intra-class correlation coefficient (ICC) with 95% confidence intervals (CI).

#### 2. Venous contrast comparison

Venous contrast of SWI_s1_, SWI_s2_, SWI_s3_ and SWI_c_ images were compared using contrast-to-noise ratio (CNR) and signal-to-noise ratio (SNR) which were calculated based on region of interest (ROI) measurement using Medical Image Processing, Analysis, and Visualization (MIPAV) software.

The CNR is defined as follows [23]:

$$CNR=\frac{\left|S_{a}-S_{b}\right|}{\sigma_{b}}$$ (3)

where S_a_ and S_b_ are the mean signal intensities in regions of DMV and regions of adjacent white matter tissues, and σ_b_ is standard deviation (SD) of the signal intensity in regions of adjacent white matter tissues.

The SNR is defined as follows [24]:

$$SNR=\frac{S}{\sigma }$$ (4)

where S is the mean signal intensity in regions of DMV, and σ is SD of the signal intensity in background air.

**Fig 1:**
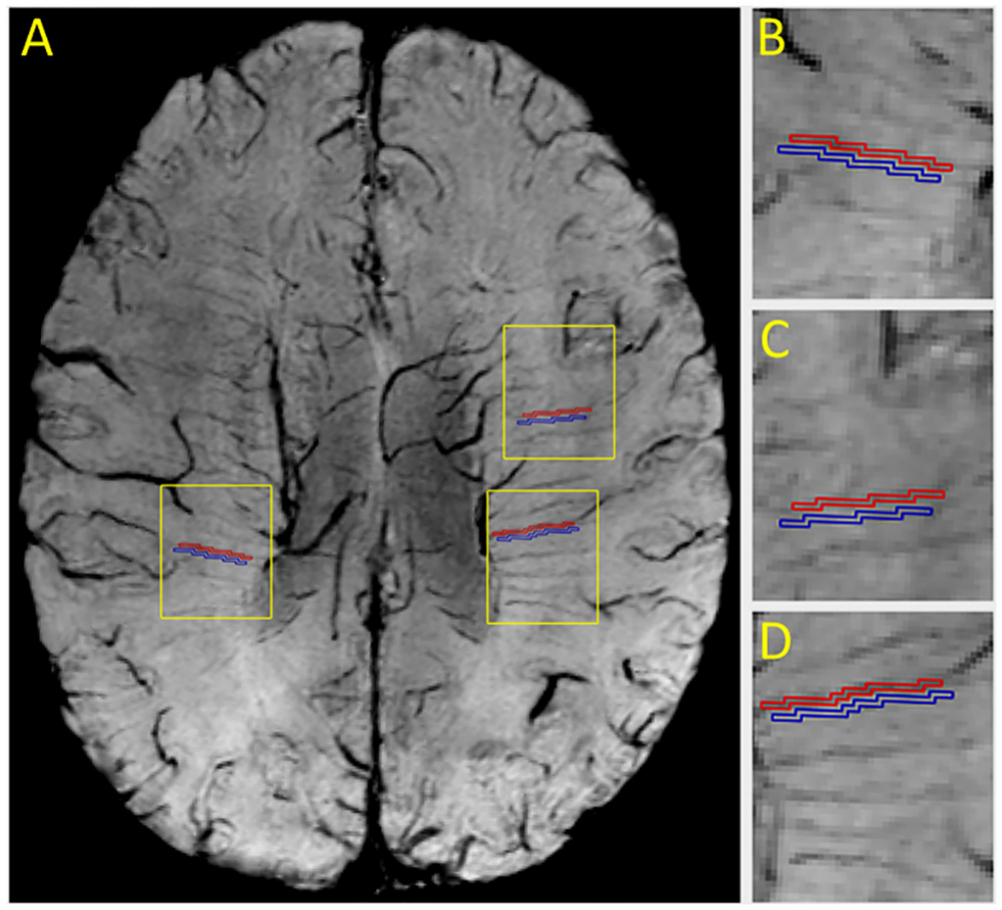
Description of contrast-to-noise ratio (CNR) measurement for deep medullary veins (DMV) in a combined multi-echo susceptibility weighted image. A, Each red region of interest (ROI) for periventricular DMV was drawn with one pixel width along the center of vein. Each blue ROI for neighboring white matter tissues was drawn surrounding the vein by the same pixel size of red ROI. Three different regions of DMV and adjacent white matter tissues (yellow rectangle in A) were shown (B to D, magnification of the three regions in A). CNR was calculated based on values obtained from the two ROIs.

Both ROIs of DMV and adjacent white matter tissues were firstly chosen in three different regions on SWI_c_ images (Fig 1) and then copied to other SWI_s_ images by an experienced radiologist. The same ROIs of DMV were used in calculating SNR. ROI was each measured again after a one-week interval by the same radiologist who was blind to previous measurements. The average results of CNR and SNR were calculated. The units of CNR and SNR are in decibels (dB) and higher CNR and SNR indicate better image quality.

### AMV evaluation

AMV was defined as increased number of DMV in one hemisphere with at least 5 more seen in comparison to the contralateral hemisphere [25]. The presence of AMV was independently evaluated by two radiologists who were blinded to the clinical data and image information using the same window level and window width by SPIN software. AMV in SWI_s1_, SWI_s2_, SWI_s3_ and SWI_c_ images were evaluated at an interval of one week. Inter-rater reliability of AMV was assessed using kappa statistics.

### Statistics

Statistical analysis was performed using SPSS 20.0 for Windows software (IBM, USA). Quantitative data are expressed as mean ± SD or median (interquartile range [IQR]), and categorical data are expressed as proportions or percentages. The significant differences in PSNR, CNR and SNR (the data were normally distributed) among the four types of SWI images were analyzed using one-way ANOVA followed by least significant difference (LSD) post-hoc analysis for multiple comparisons. The significant difference in MOS (the data were not normal distribution) among the four types of SWI images was analyzed using Kruskal-Wallis test followed by all pairwise multiple comparisons. The correlation between PSNR and MOS was performed using Spearman's correlation analysis. A two-tailed value of P < 0.05 was considered significant.

## Results

### Patient characteristics

The mean patient age was 56.2 ± 11.5 years (range, 36 to 78 years) with 14/20 (70%) being male patients. The time between MR examination and symptom onset was 4.6 ± 1.3 days (range, 3 to 7 days). NIHSS on admission was 9.1 ± 3.7 (range, 5 to 17). The detail information of stroke patients was shown in Table 1.

**Table 1:** The clinical characteristics of stroke patients.

Patient NO.	Age (yr)	Gender	Time to MRI (day)	Affected hemisphere	Lesion size (cm^3^)	NIHSS score
01	58	M	3	R	40.9	6
02	54	M	3	L	102.2	7
03	69	F	4	L	17.8	16
04	36	M	3	R	15.2	7
05	78	M	6	L	3.3	9
06	72	F	4	R	30.2	12
07	58	F	4	L	14.5	8
08	64	M	4	L	16.8	8
09	45	M	4	L	26.0	6
10	48	M	4	L	9.2	5
11	51	M	6	L	46.3	17
12	57	F	6	R	2.1	5
13	75	M	5	R	3.9	10
14	46	F	4	L	2.0	5
15	58	M	7	R	2.3	6
16	38	M	4	L	29.8	11
17	46	M	3	L	29.3	16
18	57	F	7	R	46.8	8
19	53	M	5	R	62.3	10
20	61	M	5	R	2.2	9

MRI, magnetic resonance imaging; NIHSS, National Institute of Health Stroke Score; M, male; F, female; R, right; L, left.

### Image quality and venous contrast comparison

**Fig 2:**
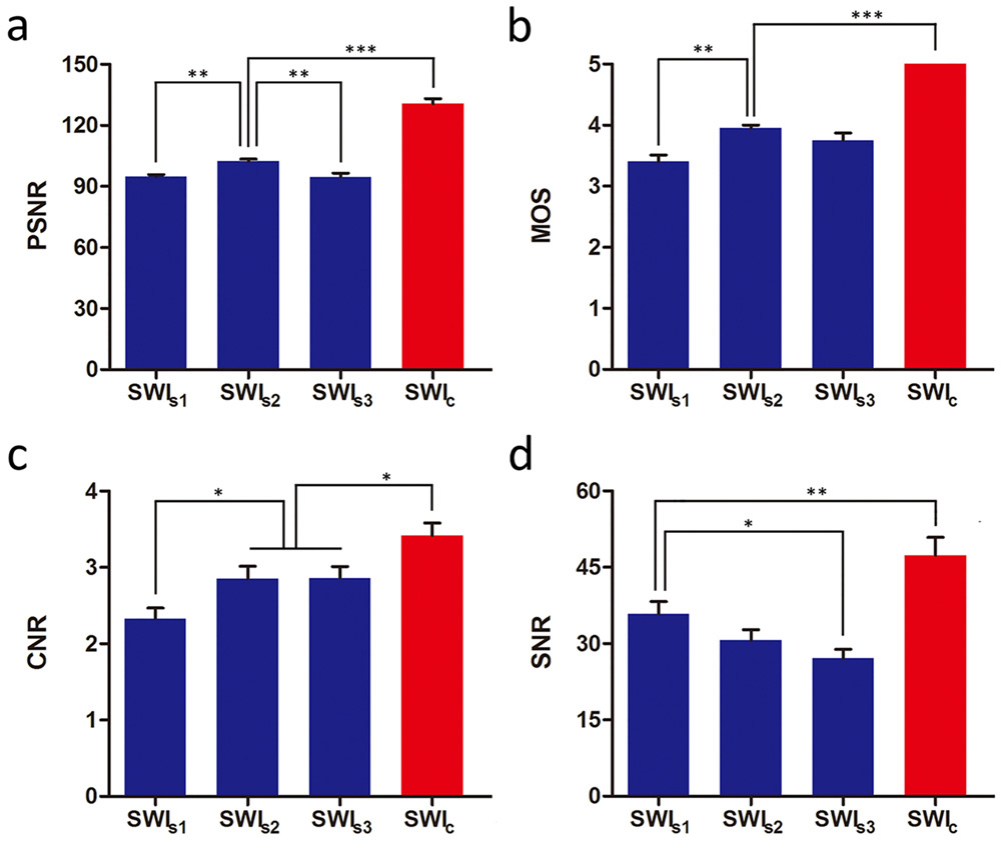
Comparisons of peak signal-to-noise ratio (PSNR), mean opinion score (MOS), contrast-to-noise ratio (CNR) and signal-to-noise ratio (SNR) between three different echoes of single-echo susceptibility weighted imaging (SWI_s_) images (blue histogram, SWI_s1_ represented TE = 23.144 ms, SWI_s2_ represented TE = 29.192 ms, SWI_s3_ represented TE = 35.240 ms) and combined multi-echo SWI images (red histogram, SWI_c_ represented combined multi-echo SWI). Note. *** indicated P < 0.001; ** indicated P < 0.01; * indicated P < 0.05. Horizontal line represented the two groups had the same value when rounding to one decimal.

SWI _s2_ had the highest PSNR value among the three different echoes of SWI_s_ (102.3 ± 5.2 dB in SWI_s2_ versus 94.8 ± 4.3 dB in SWI_s1_, P = 0.002; and versus 94.7 ± 7.9 dB in SWI_s3_, P = 0.002). There were no significant differences in PSNR value between SWI_s1_ and SWI_s3_ (P = 0.964). PSNR value of SWI_c_ was 130.9 ± 10.9 dB, increasing by 27.9% compared with PSNR value of SWI_s2_ (P < 0.001). Fig 2a showed the result of PSNR among the four types of SWI images.

The median values of MOS in SWI_s1_, SWI_s2_ and SWI_s3_ were 3 (IQR, 3–4), 4 (IQR, 4–4) and 4 (IQR, 3–4), respectively. No significant differences were found between SWI_s1_ and SWI_s2_ (P = 0.097), SWI_s1_ and SWI_s3_ (P = 0.746) and SWI_s2_ and SWI_s3_ (P = 1.000). The median values of MOS in SWI_c_ was 5 (IQR, 5–5), which was much higher than MOS of SWI_s2_ (P < 0.001). Inter-rater reliability for MOS was excellent with an ICC of 0.90 (95% CI, 0.86 to 0.93). Fig 2b showed the result of MOS among the four types of SWI images. There was a highly significant correlation between MOS and PSNR (r = 0.69, P < 0.001).

Both values of CNR in SWI_s2_ and SWI_s3_ were 2.9 ± 0.7 dB, which were significantly higher than that in SWI_s1_ (P = 0.018). CNR values of SWI_c_ was 3.4 ± 0.7 dB, increasing by 17.2% compared with CNR value of SWI_s2_ or SWI_s3_ (P = 0.013). With TE increasing, the SNR decreased. SWI_s1_ had the highest SNR value compared with SWI_s2_ and SWI_s3_ (35.8 ± 10.9 dB in SWI_s1_ versus 30.6 ± 9.4 dB in SWI_s2_, P = 0.151; and versus 27.1 ± 8.1 dB in SWI_s3_, P = 0.018). SNR value of SWI_c_ was 47.4 ± 15.8 dB, increasing by 32.4% compared with SNR value of SWI_s1_ (P = 0.002). Fig 2c and 2d showed the comparison of CNR and SNR among the four types of SWI images.

The raw data of PSNR, MOS, CNR and SNR are within S1 Dataset.

### AMV evaluation

**Fig 3:**
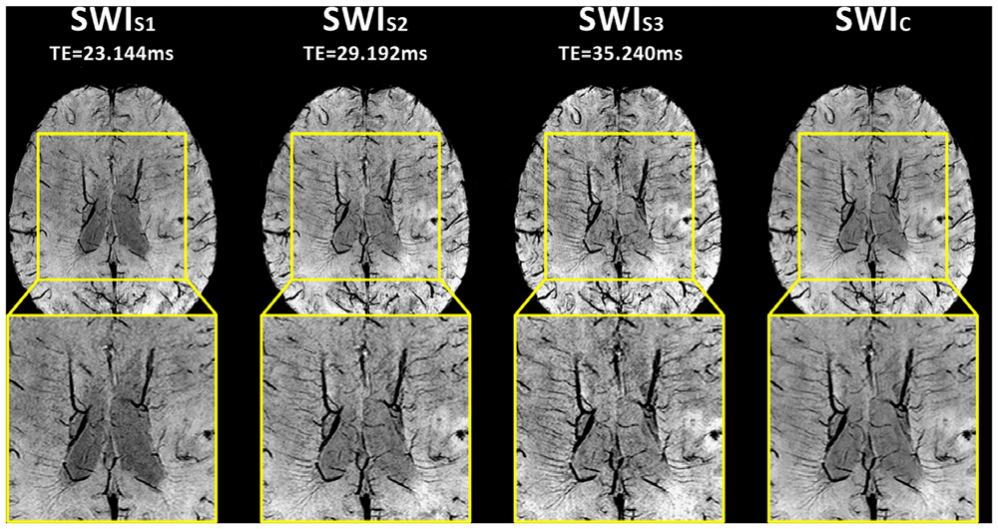
Evaluation of asymmetric medullary veins (AMV) in single-echo and combined multi-echo susceptibility weighted imaging (SWI) images. Contrast between veins and adjacent white matter tissues was better visible in the combined multi-echo SWI (SWI_c_) image than different echoes of single-echo SWI (SWI_s_) images. Detection of AMV in SWI_s2_ (TE = 29.192 ms) and SWI_s3_ (TE = 35.240 ms) was the same as that in SWI_c_. However, AMV was ambiguous in SWI_s1_ (TE = 23.144 ms).

The presence of AMV was found in 11 patients (55%) in SWI_s2_, SWI_s3_ and SWI_c_, but in 10 patients (50%) in SWI_s1_. The Kappa value of AMV was 0.80 for SWI_s1_, 0.90 for SWI_s2_, SWI_s3_ and SWI_c_. AMV were much more conspicuous in SWI_c_ images, but were inconspicuous in SWI_s1_. The case without obvious AMV in SWI_s1_ was shown in Fig 3.

## Discussion

Our study showed that TE = 29.192 ms was the optimal echo in visualizing DMV among the three different echoes of SWI_s_. Compared with SWI_s_ images, SWI_c_ images can effectively improve both image quality and venous contrast. For qualitative evaluation of AMV, SWI_s_ (except TE = 23.144 ms) had the same capability as SWI_c_.

AMV is considered to be caused by an increase of oxygen extraction within veins, due to the elevated ratio of deoxyhemoglobin to oxyhemoglobin in ischemic brain regions [26, 27]. T2*-weighted imaging (T2*WI) which is based on the blood oxygenation level dependent effect is the main method in evaluating AMV in stroke patients during hyperacute or acute stage [9, 12, 28]. SWI is developed as an alternative to T2*WI and is typically acquired at higher spatial resolution than T2*WI [1]. SWI has been confirmed to be more sensitive in detecting small paramagnetic materials than T2*WI [29–31]. Therefore, SWI should be more suitable in visualizing AMV.

SWI images using longer echoes can produce higher venous contrast. Thus, we used the latter three echoes from multi-echo SWI sequence including TE = 23.144 ms, TE = 29.192 ms and TE = 35.240 ms to reconstruct different single echoes of SWI images and found that SNR decreased when TE was prolonged. In other words, it was not the longest TE that had the best venous contrast. This result is consistent with previous finding that a longer TE has a higher venous contrast but contains severe artifacts caused by the off-resonance effect [32]. Unlike veins parallel to the B_0_ field, venous orientation of DMV with θ = 90° would be mainly influenced by an additional extravascular field around the vessel which brings additional dephasing [33]. We found that the best image quality and optimal venous contrast of DMV occurred at TE = 29.192 ms. This TE was close to the optimal time (28 ms) for veins running parallel to the main field [15, 16]. The conclusion of similar TE value producing optimum contrast for veins parallel and perpendicular to B_0_ is in accordance with an in vivo and simulation study that showed maximum contrast of venous orientation of θ = 90° was close to that of venous orientation of θ = 0° when using a comparable TE [34]. It suggests that cerebral veins, regardless of whether they are perpendicular or parallel to magnetic field, can be achieved with a high venous contrast at TE around 29 ms.

By combining SWI_s_ images acquired at different echo times, venous contrast would be improved because different contrasts of veins and susceptibility artifacts in the same zone of interest are weighted [17]. In our study, we found that SWI_c_ had a more desirable balance between contrast of small DMV and image quality and quantitative image analysis showed that image quality and venous contrast were significantly increased on SWI_c_. As far as we known, no studies have quantitatively investigated these small veins in SWI images. The main reason is that it is difficult to segment DMV because of low local contrasts and high noise levels of DMV in current MR technique. Venous contrast could be improved by using complex post-processing techniques, which has been reported in recent studies [23, 35]. The small DMV may be successfully segmented by using post-processing methods, which requires further investigation.

Although SWI_c_ showed superior contrast compared to SWI_s_, our data showed that the detection ratio of AMV in SWI_c_ images was the same as that in SWI_s_ images when using TE = 29.192 ms or 35.240 ms. The requirement for image quality in detecting the presence of AMV is not such rigorous as that in quantitative measurement of DMV because only the asymmetric performance of DMV is needed to be identified. Thus, for hyperacute or acute stage of stroke with limited scanning time or for some institutions without multi-echo SWI technique, SWI_s_ can be recommended to evaluate the presence of AMV. Based on our finding that best image quality and venous contrast of DMV was achieved at TE = 29.192 ms, a SWI_s_ with a relative long TE (around 29 ms) seems to be sufficient. However, using a shorter TE in SWI_s_ should be cautious because it may underestimate the presence of AMV. Small vessels at shorter TE have a lower venous contrast, and therefore, DMV may be difficult to be differentiated from adjacent image noise.

Image resolution is also an important issue in delineating intracranial small veins. Due to the normal caliber of DMV ranging from 100 μm to 250 μm [36], a high image resolution is necessary. It has been known that high in-plane resolution with a thicker slice is best for SWI images. The concept of voxel aspect ratio, defined as a ratio of R/w/h (R is the diameter of the vein, and w and h are the width and height of the voxel dimension), has been reported to play an important role in venous contrast of SWI images [37]. It is suggested that a voxel aspect ratio of 1:1:4 is optimal for venous contrast of a vein perpendicular to the main field [37]. In our study, an in-plane resolution of 0.47 mm × 0.47 mm with a slice thickness of 2 mm was used. Although DMV are smaller than the voxel dimensions, a resolution of 0.5 mm × 0.5 mm with a 2 mm slice thickness still can yield a good venous contrast for veins with diameter of roughly 200 μm or greater [38].

There are some limitations in our study. Firstly, an additional single-echo sequence was not acquired for comparison. Different echoes of SWI_s_ images derived from a multi-echo sequence has its own advantage that all parameters are the same besides TE. In fact, it is difficult to obtain the same sequence parameters between a reference SWI_s_ image and a SWI_c_ image because some parameters have to be adjusted to allocate time for the sampling of multiple echoes. Secondly, the maximum TE was only 35.240 ms. It is unknown whether combined with other TEs of longer than 35.240 ms would produce a better image quality and venous contrast. However, a too long TE will increase the total scan time, which is not practicable in the clinical setting of hyperacute stroke when “time is brain” [39].

In summary, SWI_s_ using TE around 29 ms was the optimal echo for visualizing AMV in stroke patients. SWI_c_ could improve visibility of AMV, but was equal to SWI_s_ using a relative long TE in qualitative assessment of AMV. These results will provide the technique basis for choosing the optimal echo for visualizing AMV and pave the way for further research of AMV in stroke.

## Supporting Information

S1 Dataset (XLSX)

## References

[1] Haacke EM, Xu Y, Cheng YCN, Reichenbach JR. Susceptibility weighted imaging (SWI). Magn Reson Med. 2004;52: 612–618. 15334582 10.1002/mrm.20198

[2] Haacke E, Mittal S, Wu Z, Neelavalli J, Cheng YC. Susceptibility-weighted imaging: technical aspects and clinical applications, part 1. AJNR Am J Neuroradiol. 2009;30: 19–30. 10.3174/ajnr.A1400 19039041 PMC3805391

[3] Mittal S, Wu Z, Neelavalli J, Haacke E. Susceptibility-weighted imaging: technical aspects and clinical applications, part 2. AJNR Am J Neuroradiol. 2009;30: 232–252. 10.3174/ajnr.A1461 19131406 PMC3805373

[4] Xu X, Wang Q, Zhang M. Age, gender, and hemispheric differences in iron deposition in the human brain: an in vivo MRI study. Neuroimage. 2008;40: 35–42. 10.1016/j.neuroimage.2007.11.017 18180169

[5] Yan SQ, Sun JZ, Yan YQ, Wang H, Lou M. Evaluation of brain iron content based on magnetic resonance imaging (MRI): comparison among phase value, R2* and magnitude signal intensity. PloS one. 2012;7: e31748 10.1371/journal.pone.0031748 22363719 PMC3282752

[6] Radbruch A, Mucke J, Schweser F, Deistung A, Ringleb PA, Ziener CH, et al Comparison of susceptibility weighted imaging and TOF-angiography for the detection of Thrombi in acute stroke. PloS one. 2013;8: e63459 10.1371/journal.pone.0063459 23717426 PMC3662691

[7] Boeckh-Behrens T, Lutz J, Lummel N, Burke M, Wesemann T, Schöpf V, et al Susceptibility-weighted angiography (SWAN) of cerebral veins and arteries compared to TOF-MRA. Eur J Radiol. 2012;81: 1238–1245. 10.1016/j.ejrad.2011.02.057 21466929

[8] Horie N, Morikawa M, Nozaki A, Hayashi K, Suyama K, Nagata I. “Brush sign” on susceptibility-weighted MR imaging indicates the severity of moyamoya disease. AJNR Am J Neuroradiol. 2011;32: 1697–1702. 10.3174/ajnr.A2568 21799039 PMC7965393

[9] Terasawa Y, Yamamoto N, Morigaki R, Fujita K, Izumi Y, Satomi J, et al Brush sign on 3-T T2*-weighted MRI as a potential predictor of hemorrhagic transformation after tissue plasminogen activator therapy. Stroke. 2014;45: 274–276. 10.1161/STROKEAHA.113.002640 24172577

[10] De Guio F, Vignaud A, Ropele S, Duering M, Duchesnay E, Chabriat H, et al Loss of venous integrity in cerebral small vessel disease: A 7-T MRI study in cerebral autosomal-dominant arteriopathy with subcortical infarcts and leukoencephalopathy (CADASIL). Stroke. 2014;45: 2124–2126. 10.1161/STROKEAHA.114.005726 24867926

[11] Yu X, Yuan L, Jackson A, Sun J, Huang P, Xu X, et al Prominence of medullary veins on susceptibility-weighted images provides prognostic information in patients with subacute stroke. AJNR Am J Neuroradiol. 2016;37: 423–429. 10.3174/ajnr.A4541 26514606 PMC7960117

[12] Morita N, Harada M, Uno M, Matsubara S, Matsuda T, Nagahiro S, et al Ischemic findings of T2*-weighted 3-tesla MRI in acute stroke patients. Cerebrovasc Dis. 2008;26: 367–375. 10.1159/000151640 18728364

[13] Jensen-Kondering U, Böhm R. Asymmetrically hypointense veins on T2*w imaging and susceptibility-weighted imaging in ischemic stroke. World J Radiol. 2013;5: 156–164. 10.4329/wjr.v5.i4.156 23671751 PMC3647207

[14] Santhosh K, Kesavadas C, Thomas B, Gupta A, Thamburaj K, Kapilamoorthy TR. Susceptibility weighted imaging: a new tool in magnetic resonance imaging of stroke. Clin Radiol. 2009;64: 74–83. 10.1016/j.crad.2008.04.022 19070701

[15] Reichenbach JR, Barth M, Haacke EM, Klarhöfer M, Kaiser WA, Moser E. High-resolution MR venography at 3.0 Tesla. J Comput Assist Tomogr. 2000;24: 949–957. 11105717 10.1097/00004728-200011000-00023

[16] Koopmans PJ, Manniesing R, Niessen WJ, Viergever MA, Barth M. MR venography of the human brain using susceptibility weighted imaging at very high field strength. Magn Reson Mater Phy. 2008;21: 149–158. 10.1007/s10334-007-0101-3 18188626

[17] Denk C, Rauscher A. Susceptibility weighted imaging with multiple echoes. J Magn Reson Imaging. 2010;31: 185–191. 10.1002/jmri.21995 20027586

[18] Du YP, Jin Z. Simultaneous acquisition of MR angiography and venography (MRAV). Magn Reson Med. 2008;59: 954–958. 10.1002/mrm.21581 18429022

[19] Mucke J, Möhlenbruch M, Kickingereder P, Kieslich PJ, Bäumer P, Gumbinger C, et al Asymmetry of Deep Medullary Veins on Susceptibility Weighted MRI in Patients with Acute MCA Stroke Is Associated with Poor Outcome. PloS one. 2015;10: e0120801 10.1371/journal.pone.0120801 25849958 PMC4388537

[20] Tanchenko A. Visual-PSNR Measure of Image Quality. J Vis Commun Image Represent. 2014;25: 874–878.

[21] Cosman PC, Gray RM, Olshen RA. Evaluating quality of compressed medical images: SNR, subjective rating, and diagnostic accuracy. Proceedings of the IEEE. 1994;82: 919–932.

[22] Ghrare S, Ali M, Ismail M, Jumari K. Diagnostic quality of compressed medical images: objective and subjective evaluation. In Second Asia international conference on modeling and simulation; 2008. IEEE. pp. 923–927.

[23] Jang U, Nam Y, Kim DH, Hwang D. Improvement of the SNR and resolution of susceptibility-weighted venography by model-based multi-echo denoising. Neuroimage. 2013;70: 308–316. 10.1016/j.neuroimage.2012.12.067 23296184

[24] Dietrich O, Raya JG, Reeder SB, Reiser MF, Schoenberg SO. Measurement of signal-to-noise ratios in MR images: Influence of multichannel coils, parallel imaging, and reconstruction filters. J Magn Reson Imaging. 2007;26: 375–385. 17622966 10.1002/jmri.20969

[25] Horie N, Morikawa M, Nozaki A, Hayashi K, Suyama K, Nagata I. “Brush sign” on susceptibility-weighted MR imaging indicates the severity of moyamoya disease. AJNR American Journal of Neuroradiology. 2011;32: 1697–1702. 10.3174/ajnr.A2568 21799039 PMC7965393

[26] Tamura H, Hatazawa J, Toyoshima H, Shimosegawa E, Okudera T. Detection of deoxygenation-related signal change in acute ischemic stroke patients by T2*-weighted magnetic resonance imaging. Stroke. 2002;33: 967–971. 11935045 10.1161/01.str.0000013672.70986.e2

[27] Kesavadas C, Santhosh K, Thomas B. Susceptibility weighted imaging in cerebral hypoperfusion—can we predict increased oxygen extraction fraction? Neuroradiology. 2010;52: 1047–1054. 10.1007/s00234-010-0733-2 20567811

[28] Rosso C, Belleville M, Pires C, Dormont D, Crozier S, Chiras J, et al Clinical usefulness of the visibility of the transcerebral veins at 3T on T2*-weighted sequence in acute stroke patients. Eur J Radiol. 2012;81: 1282–1287. 10.1016/j.ejrad.2011.03.025 21444172

[29] Mori N, Miki Y, Kikuta K-i, Fushimi Y, Okada T, Urayama S-i, et al Microbleeds in moyamoya disease: susceptibility-weighted imaging versus T2*-weighted imaging at 3 Tesla. Invest Radiol. 2008;43: 574–579. 10.1097/RLI.0b013e31817fb432 18648257

[30] Cheng AL, Batool S, McCreary CR, Lauzon M, Frayne R, Goyal M, et al Susceptibility-Weighted Imaging is More Reliable Than T2*-Weighted Gradient-Recalled Echo MRI for Detecting Microbleeds. Stroke. 2013;44: 2782–2786. 10.1161/STROKEAHA.113.002267 23920014

[31] Lobsien D, Dreyer AY, Stroh A, Boltze J, Hoffmann KT. Imaging of VSOP labeled stem cells in agarose phantoms with susceptibility weighted and T2* weighted MR imaging at 3T: determination of the detection limit. PLoS One. 2013;8: e62644 10.1371/journal.pone.0062644 23667503 PMC3648551

[32] Du YP, Jin Z, Hu Y, Tanabe J. Multi-echo acquisition of MR angiography and venography of the brain at 3 Tesla. J Magn Reson Imaging. 2009;30: 449–454. 10.1002/jmri.21833 19629975

[33] Deistung A, Rauscher A, Sedlacik J, Stadler J, Witoszynskyj S, Reichenbach JR. Susceptibility weighted imaging at ultra high magnetic field strengths: theoretical considerations and experimental results. Magn Reson Med. 2008;60: 1155–1168. 10.1002/mrm.21754 18956467

[34] Sedlacik J, Rauscher A, Reichenbach JR. Obtaining blood oxygenation levels from MR signal behavior in the presence of single venous vessels. Magn Reson Med. 2007;58: 1035–1044. 17969121 10.1002/mrm.21283

[35] Quinn M, Gati J, Klassen L, Lin A, Bird J, Leung S, et al Comparison of multiecho postprocessing schemes for SWI with use of linear and nonlinear mask functions. AJNR Am J Neuroradiol. 2014;35: 38–44. 10.3174/ajnr.A3584 23744694 PMC7966483

[36] Hooshmand I, Rosenbaum A, Stein R. Radiographic anatomy of normal cerebral deep medullary veins: criteria for distinguishing them from their abnormal counterparts. Neuroradiology. 1974;7: 75–84. 4369105 10.1007/BF00341874

[37] Xu Y, Haacke EM. The role of voxel aspect ratio in determining apparent vascular phase behavior in susceptibility weighted imaging. Magn Reson Imaging. 2006;24: 155–160. 16455403 10.1016/j.mri.2005.10.030

[38] Reichenbach JR, Haacke EM. High-resolution BOLD venographic imaging: a window into brain function. NMR Biomed. 2001;14: 453–467. 11746938 10.1002/nbm.722

[39] Jauch EC, Saver JL, Adams HP, Bruno A, Demaerschalk BM, Khatri P, et al Guidelines for the early management of patients with acute ischemic stroke: a guideline for healthcare professionals from the American Heart Association/American Stroke Association. Stroke. 2013;44: 870–947. 10.1161/STR.0b013e318284056a 23370205

